# Characteristics and topic trends on electrical impedance tomography hardware publications

**DOI:** 10.3389/fphys.2022.1011941

**Published:** 2022-10-13

**Authors:** Shaojie Qin, Yulong Yao, Yuqing Xu, Danling Xu, Yuan Gao, Shunpeng Xing, Zhe Li

**Affiliations:** Department of Critical Care Medicine, Renji Hospital, School of Medicine, Shanghai Jiao Tong University, Shanghai, China

**Keywords:** bibliometric analysis, EIT, hardware, WoSCC, hotspot

## Abstract

**Objective:** Electrical impedance tomography (EIT) is a technique to measure electrical properties of tissue. With the progress of modern integrated circuits and microchips, EIT instrumentation becomes an active research area to improve all aspects of device performance. Plenty of studies on EIT hardware have been presented in prestigious journals. This study explores publications on EIT hardware to identify the developing hotspots and trends.

**Method:** Publications covering EIT hardware on the Web of Science Core Collection (WoSCC) database from 1989 to 2021 were collected for bibliometric analysis. CiteSpace and VOS viewer were used to study the characteristics of the publications.

**Main results:** A total of 592 publications were analyzed, showing that the number of annual publications steadily increased. China, England, and South Korea were the most prolific countries on EIT hardware publications with productive native institutions and authors. Research topics spread out in “bio-electrical impedance imaging”, “hardware optimization”, “algorithms” and “clinical applications” (e.g., tissue, lung, brain, and oncology). Hardware research in “pulmonary” and “hemodynamic” applications focused on monitoring and were represented by silhouette recognition and dynamic imaging while research in “tumor and tissue” and “brain” applications focused on diagnosis and were represented by optimization of precision. Electrode development was a research focus through the years. Imaging precision and bioavailability of hardware optimization may be the future trend.

**Conclusion:** Overall, system performance, particularly in the areas of system bandwidth and precision in applications may be the future directions of hardware research.

## Introduction

Electrical impedance tomography (EIT) is a non-invasive and radiation-free functional imaging technology with the real-time application of monitoring clinical pathophysiological changes at the bedsid (Holder, 2005). Since the introduction of the first systems in the early 1980s, EIT instrumentation has continued to evolve in step with advances in analog, digital electronics, equipment design and clinical applications (Gaggero et al., 2012; Frerichs et al., 2017; Saulnier et al., 2020). Research topics regarding EIT hardware have been presented in different professional journals and scientific conferences. However, the emerging trends and hot spots of publications in this field have not been studied.

Bibliometric analysis explores research publications based on big data and algorithms, which has an innate advantage in dealing with huge amounts of data (Aria and Cuccurullo, 2017). Therefore, bibliometric analysis is used to assess contributions and highlight the application characteristics of publications related to a research field of interest (Donthu et al., 2021). In order to explore the current stand, hot spots, and new trends of EIT hardware research, we conducted a bibliometric analysis on this subject from 1989 to 2021. This study elaborates the characteristics of hardware-related publications, identify the influential articles, provide insights for the most noteworthy research areas and predict future study directions.

## Methods

### Source database and retrieval strategies

Web of Science Core Collection (WoSCC) database is one of the most common databases used for bibliometric analysis. In this study, all data were retrieved from the WoSCC, the strategy was TI OR AB OR AK = (“electrical impedance tomography”) AND TI OR AK =((“current”) OR (“voltage”) OR (“electrode”) OR (“system”)) NOT TI=(“algorithm” or “reconstruction”); Time window: 1989.01.01 to 2021.12.31; Publication type: “Article”, “Proceeding paper”, “Review” and “Publish on line”; Language: English. All investigators collected the literature on 2022.05.12 to avoid database update bias.

### Data collection

All results were searched by two investigators independently and the agreement of the results was 98%, showing significant consistency. The data were further screened by at least two experienced experts and publications less relevant to hardware were defined as studies and reviews based on image processing, algorithm, and applications, and other disciplines like Soil Science, Polymer Science, Plant Sciences are excluded. The data were exported in a text format and a UTF-8 format for further software analysis.

### Bibliometric analysis by WoSCC output

The Web of Science-Incites Research Performance Analysis Platform (WoS-Incites, https://incites.clarivate.com/) and Microsoft Excel (version: Microsoft 365) were used to output the characteristics of publications, including the annual publication number, countries, institutions, journals, impact factors (IF), Journal Citation Reports (JCR) of the journals, citations, etc.

### Bibliometric analysis by VOS viewer

Bibliometric software VOS viewer (VOS, 1.6.16, Leiden University, Leiden, the Netherlands) was used to analyze and visualize co-authorship of authors, institutions, countries, and keywords network. The relevant thresholds were as follows: keywords (occurrence>1 or 5), collaborative of institutions (publications>2), authors (publications>1), and countries (publications>1). To improve the efficiency of analysis, we merged keywords that have different expressions but the same meaning, merge details were available in Supplementary Table S1.

### Bibliometric analysis by CiteSpace

Bibliometric software CiteSpace 5.8R3 (Chen Meichao, Drexel University) was used to complete keyword burst analysis.

### Statistical analysis

Continuous variables were expressed as mean ± standard deviation (mean ± SD) or median (Interquartile range) [M(IQR)] depending on whether they followed normal distribution. Categorical variables were described using cases and percentages or proportions. And differences between groups were compared by chi-square test. A two-sided *p* values less than 0.05 were considered statistically significance.

## Results

### Bibliometric analysis of publication output

**FIGURE 1:**
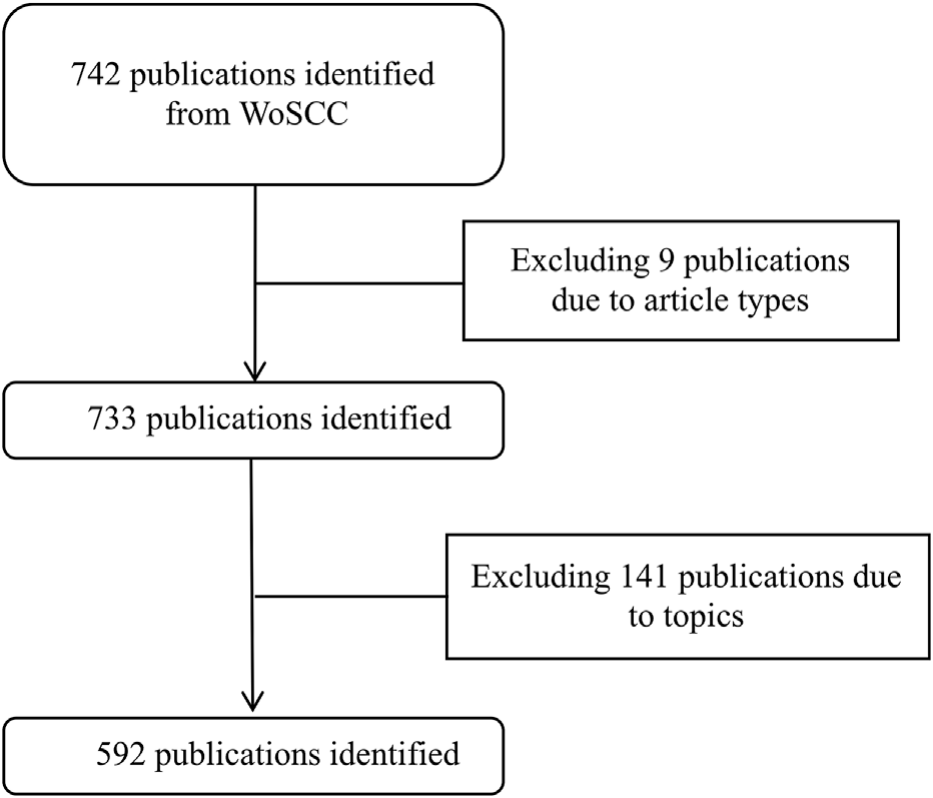
Flow chart of included publications. WoSCC, Web of science center.

As to the retrieval strategy, 742 publications were identified and extracted from WoSCC. Finally, a total of 592 research articles, reviews, letters, and proceeding papers published in English on EIT hardware were analyzed (Figure 1).

### Growth trend and quality of global publications on EIT hardware

**FIGURE 2:**
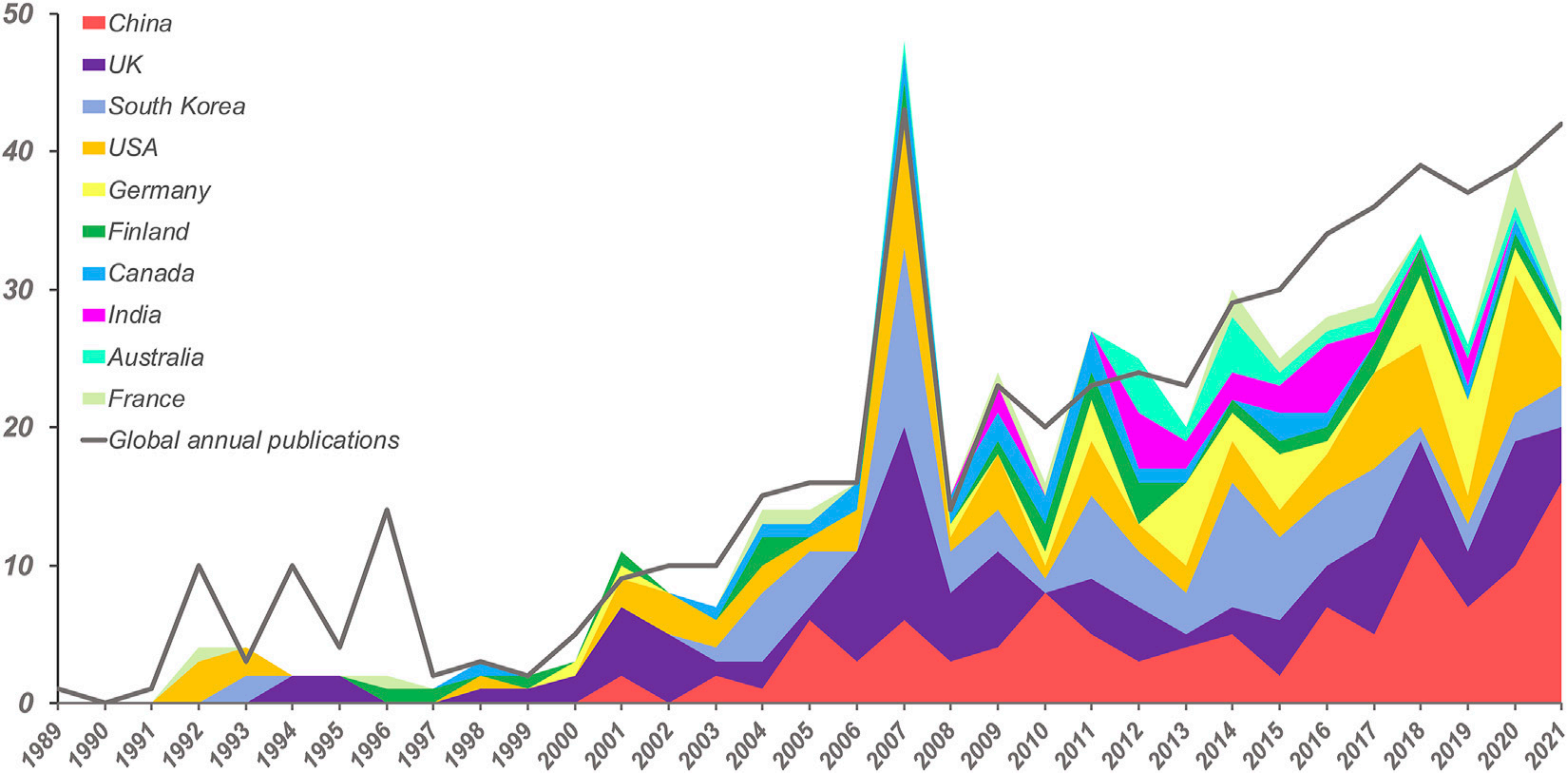
Annual growth trends of publications by global and top 10 countries on electrical impedance tomography hardware research from 1989.01.01 to 2021.12.31. The grey line indicates the trend of annual world publications, the colors indicate the top 10 most productive countries. The width of the color plate indicates the number of publications.

The global and national growth trend of EIT hardware publication from 1989 to 2021 illustrated the temporal and geographic productivity. The annual publications numbers of the global and top 10 countries were shown in Figure 2. Global EIT publications increased generally, especially after 2000. The year 2007 is a phased peak of EIT hardware researches and the publications exploded since then. Up to now, 39 countries have published literatures on EIT hardware. The most types of publications are Article (76.7%) and Proceeding paper (20.3%). Among these, China, England, South Korea, the USA, and Germany ranked as the leading five prolific contributors, respectively taking up 18.91%, 17.89%, 13.29%, 13.12%, and 6.13% of the total number of publications. In addition, China started late in the year 2001 but increased rapidly after that.

Books and reviews have the highest average citations. Stratified with the IF, 454(8.02%), 81(70.75%), 23 (18.00%) and 34 (5.7%) out of 592 publications were published in journals with IF ≤ 5, IF between 5 and 10 (5 < IF < 10), IF ≥ 10 and without IF, respectively. Notably, there is no difference in the average citations across each IF segments. In contrast, average citations of studies published in the journal with JCR Q1 and JCR Q2 were significantly higher compared with JCR Q3 and JCR Q4 (Table 1).

**TABLE 1:** Quality of EIT hardware publications.

	N (proportion%)	Average citation	*p* Value
Publication type^a^			
Article	454 (76.7%)	20.49	
Review	12 (2.0%)	86.89	
Proceeding Paper	120 (20.3%)	25.04	
Early Access	5 (0.8%)	1.33	
Book chapter	1 (0.2%)	279.00	
Publications in journals with IF^b^			
0＜IF ≤ 5	454 (76.7%)	21.08
5＜IF＜10	81 (13.7%)	15.42
IF ≥ 10	23 (3.9%)	24.80	*p* > 0.05
N\A	34 (5.7%)	23.27
Publications in journals with JCR^c^			
Q1	221 (37.3%)	20.25
Q2	274 (46.3%)	24.97
Q3	45 (7.6%)	8.10	*p* ＜ 0.01
Q4	21 (3.5%)	4.50
N\A	31 (5.2%)	13.05

a Publication type: There were cross-over among type “Article, Proceeding Paper, Early Access” and between type “Review, Book chapter”.

b IF, impact factor.

c JCR, journal citation reports.

### Academic actualities in the research field of EIT hardware

The top 10 most productive institutions, journals, authors, and most cited references on EIT hardware research are summarized in Table 2. The majority of the most contributed institutions (described as abbreviation in the software delivered results; number of publications; citing frequency) were universities. Among them, three are from South Korea, three from the UK, and two from China. The Kyung Hee University (Kyung Hee Univ; 58; 355) contributed the most publications, followed by University College London (UCL; 54; 329) and Fourth Military Medical University (Fourth Mil Med Univ; 25; 36). Meanwhile, Kyung Hee Univ also ranked first in citations. The journal of Physiological Measurement (*PHYSIOL MEAS*; 100; 738) published most EIT hardware publications and had the largest number of total citations while “IEEE Transactions on Medical Imaging” (*IEEE T MED IMAGING*; 15; 55) has the highest impact factor (IF). So far, 142 authors have contributed to EIT hardware publications. Overall, Woo, EJ (42; 267) was the most productive author, followed by Holder, DS (32; 212) and Oh, TI (26; 180). The 10 reference articles with the highest citation consisted of two reviews, seven original articles, and one proceeding paper. “Somersalo, E: Existence and uniqueness for electrode models for electric current computed tomography. SIAM J. APPL. MATH, 1992 (Somersalo et al., 1992).” was the reference article with the highest cited frequency. Details of the top 10 cited reference articles on EIT hardware publications were listed in Supplementary Table S2.

**TABLE 2:** The top 10 institutions, journals, authors and cited reference articles on EIT hardware research.

**In stitu tion**	**Publica tions**	**Cita tions**	**Journal**	**Publica tions**	**Cita tions**	**IF**	**Author**	**Publica tions**	**Cita tions**	**Cited References**	**Cita tions**
Kyung Hee Univ	58	355	PHYSIOL MEAS	100	738	2.833	Woo, EJ	42	267	Somersalo et al., 1992	585
UCL	54	329	MEAS SCI TECHNOL	22	96	2.046	Holder, DS	32	312	Bayford et al., 2006	259
Fourth Mil Med Univ	25	36	IEEE T BIO-MED ENG	18	151	4.538	Oh, TI	26	180	Vauhkonen et al., 1999	245
Rensselaer Polytech Inst	24	186	IEEE T MED IMAGING	15	55	10.048	Bayford, R	24	188	Cherepenin et al., 2001	156
Middlesex Univ	22	180	IEEE SENS J	15	47	3.301	Demosthenous, A	18	67	Woo et al., 2008	154
Dartmouth Coll	21	146	IEEE T BIOMED CIRC S	12	115	3.833	Dong, XZ	17	36	Halter et al., 2008	123
Konkuk Univ	20	84	IEEE T INSTRUM MEAS	11	27	4.016	Kwon, OI	17	64	McAdams et al., 1996	105
Cheju Natl Univ	20	8	13th ICEBI(ISCAS)	9	12	Q	Fu, F	16	25	Cherepenin et al., 2002	104
Univ Sydney	16	65	WC2006	9	5	Q	Adler, A	15	94	Kolehmainen et al., 1997	102
Tianjin Univ	16	35	SIAM J APPL MATH	8	84	2.08	Shi, XT	14	32	Wu Y et al., 2018	99

IF, impact factor.

### Cooperation between countries, authors, and institutions on EIT hardware research

**FIGURE 3:**
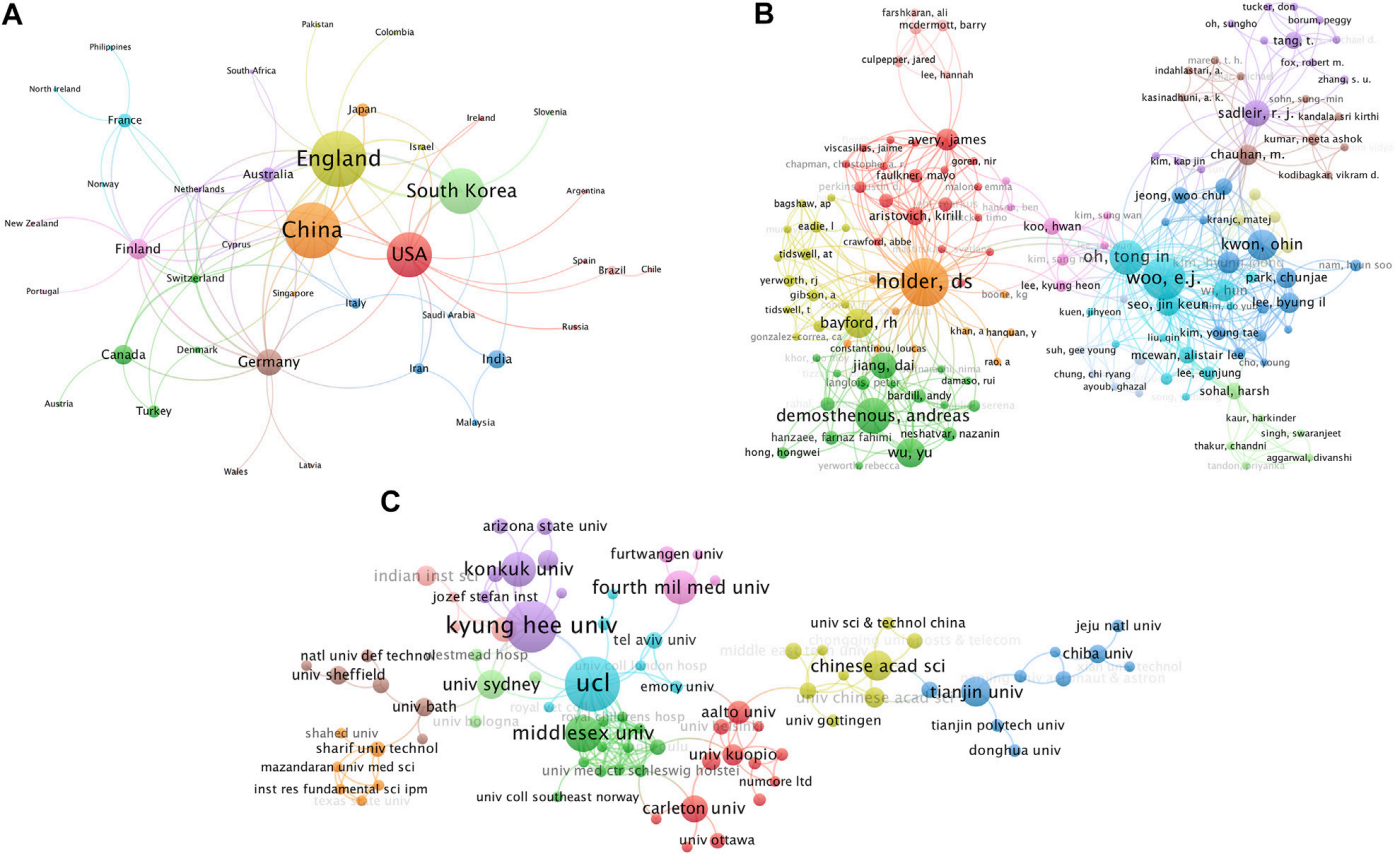
Cooperation of countries **(A)**, authors **(B)**, and institutions **(C)** on electrical impedance tomography hardware research from 1989.01.01 to 2021.12.31. The different colors represent countries **(A)**, authors **(B)**, and institutions **(C)**; color plate size indicates the number of publications, respectively; line thickness indicates the strength of cooperation.

Visualization maps of cooperation between countries, authors, and institutions on EIT hardware research were shown in Figure 3. Close collaboration (described as total link strength) can be observed in the most productive countries, institutions, and authors. England (42), Germany (28), and the USA (27) hold the most active cooperation with other countries. Meanwhile, In the largest author collaboration network of 142 authors, Woo, EJ (117), Holder, DS (116), and OH, TI (87) are the most cooperative authors. UCL (51), Kyung Hee Univ (46), and Middlesex Univ (38) are the three most collaborative institutions.

### Topic hotspot and trend of EIT hardware research

**FIGURE 4:**
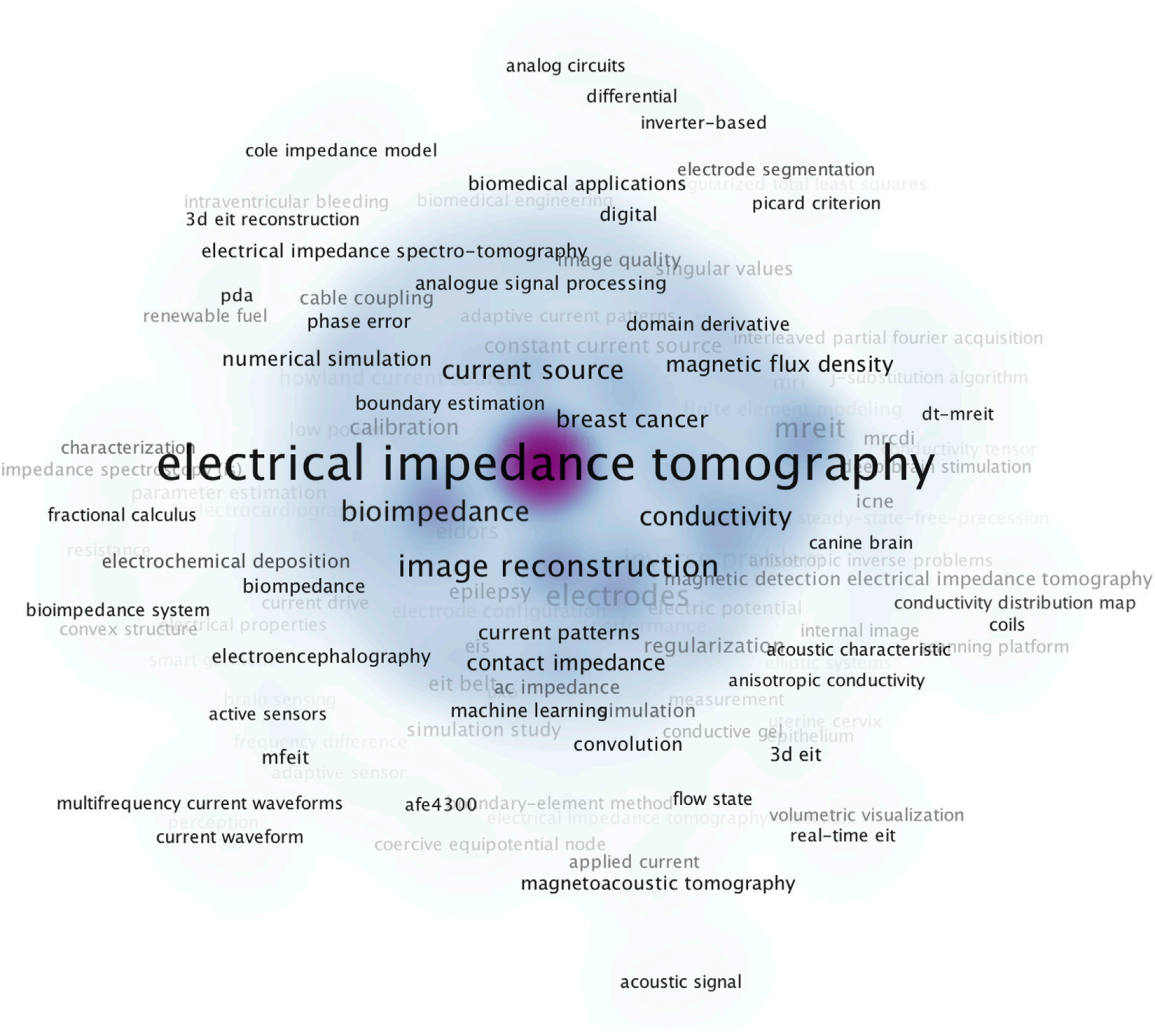
Visualization map of keywords density on EIT hardware research from 1989.01.01 to 2021.12.31. The label indicates keywords, the reduced color from purple to white indicates density of each keyword calculated by frequency of co-occurrence.

A total of 1,181 keywords were extracted from the collected 592 articles, the occurrence density calculated by frequency of co-occurrence was visualized in Figure 4, and the keywords with high co-occurrence density were focus on major topic of “bioimpedance”, “conductivity”, “current source” while the keywords regarding hardware details as “partial Fourier acquisition”, “system performance”, “wireless” were with less co-occurrence density.

**FIGURE 5:**
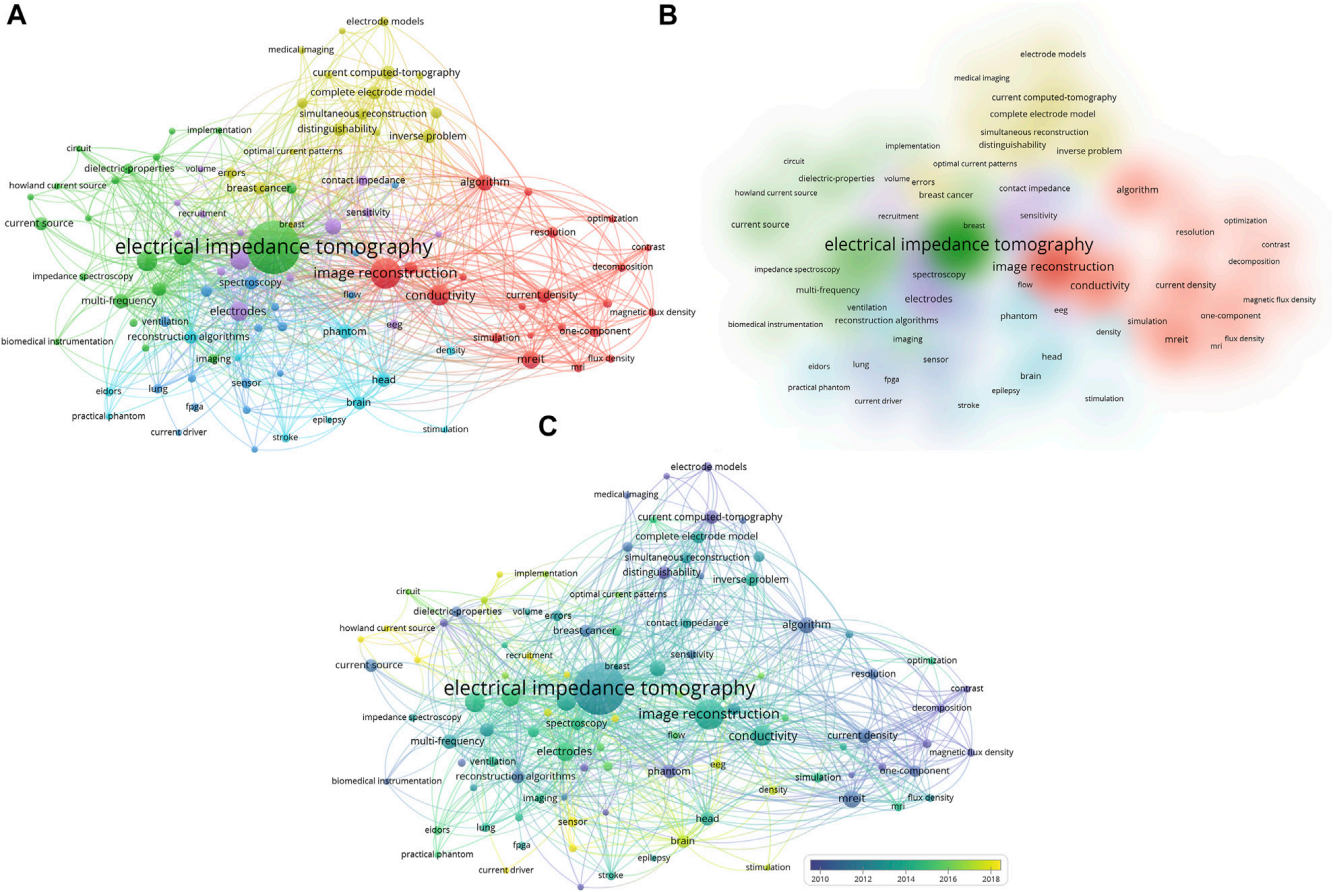
Visualization map of keywords co-occurrence network **(A,B)** and overlay **(C)** analysis on EIT hardware research from 1989.01.01 to 2021.12.31. The color indicates each cluster **(A,B)** and the average publication year **(C)**, due to the limited number of publications before 2010, they share the same color as 2010 **(C)**. The circle size indicates frequency of co-occurrences **(A,C)**. The line thickness indicates link strengths **(A–C)**.

Further, 101 keywords that met the threshold of co-occurrence frequency five or more times were added to the co-occurrence network and overlay analysis. The keywords co-occurred in 6 clusters (Figures 5A,B), beside the cluster regarding “bio-electrical impedance imaging” and “hardware optimization”, the other clusters spread out in “algorithms”, “clinical applications” (e.g.*,* tissue, lung, brain, and oncology). The similar situation also appears in the keywords with high co-occurrence frequency, besides the search terms “electrical impedance tomography” and “electrode”, the top five highly co-occurrence keywords (described as occurrence frequency; total link strengths) were “image reconstruction” (127; 556), “conductivity” (56; 324), “design” (53; 204), “bioimpedance” (42; 131) and “mreit” (22; 61). The top 25 highly co-occurrence keywords are listed in Table 3. Three of them were related to algorithm, which are “image reconstruction”, “reconstruction algorithm”, and “algorithm”; and three were related to applications, which are “head”, “brain”, and “breast cancer”. Details of major keywords in each cluster are listed in Supplementary Table S3.

**TABLE 3:** Top 25 high frequency keywords on electrical impedance tomography hardware research.

Rank	Keyword	Occurrences	Link strengths
1	electrical impedance tomography	415	1,109
2	image reconstruction	127	556
3	conductivity	56	324
4	design	53	204
5	system	51	176
6	electrodes	45	158
7	bioimpedance	42	131
8	mreit	39	225
9	algorithm	31	163
10	model	31	153
11	current density	26	155
12	multi-frequency	26	113
13	calibration	23	100
14	current computed-tomography	23	80
15	inverse problem	23	92
16	phantom	23	123
17	head	22	126
18	brain	21	92
19	complete electrode model	21	77
20	reconstruction algorithms	21	101
21	current source	20	39
22	distinguishability	20	70
23	spectroscopy	20	102
24	performance	19	124
25	breast cancer	18	73

The overlay analysis of keywords represents the topic trends of EIT research in hardware. The color distribution in the overlay visual map showed different time periods (Figure 5C). The 10 earliest and latest keywords (described as co-occurrence frequency; average publication year) were summarized in Table 4. In addition to “current computed-tomography” (23; 2005.00), which was the former title of EIT, “distinguishability” (20; 2009.26) had the highest co-occurrence frequency in the earliest keywords, while “sensor” (13; 2018.92) had the highest co-occurrence frequency in the latest keywords. Notably keywords related to electrode development both appeared in both the early “electrode models” (11; 2006.70) and recent “textile electrodes” (5; 2020.00) period.

**TABLE 4:** Top 10 earliest and latest keywords on electrical impedance tomography hardware research.

	Earliest			Latest		
Rank	Keyword	Occurrences	Avg.pub.year	Keyword	Occurrences	Avg.pub.year
1	current computed-tomography	23	2005.00	voltage measurement	8	2020.75
2	electrode models	11	2006.70	textile electrodes	5	2020.00
3	cancer detection	5	2007.20	skin	6	2019.67
4	state estimation	5	2HI008.40	sensor	13	2018.92
5	decomposition	9	2009.22	active electrode	5	2018.20
6	distinguishability	20	2009.26	output impedance	5	2018.20
7	Functional imaging	5	2009.40	recruitment	6	2018.00
8	b-z algorithm	9	2009.44	current driver	5	2018.00
9	contrast	6	2009.50	Howland current source	5	2017.80
10	biological tissues	8	2009.62	magnetoacoustic tomography	5	2017.60

Avg.pub.year, the average publication year of the articles in which the keyword occurs.

### Topics of EIT hardware research in specific research applications

**FIGURE 6:**
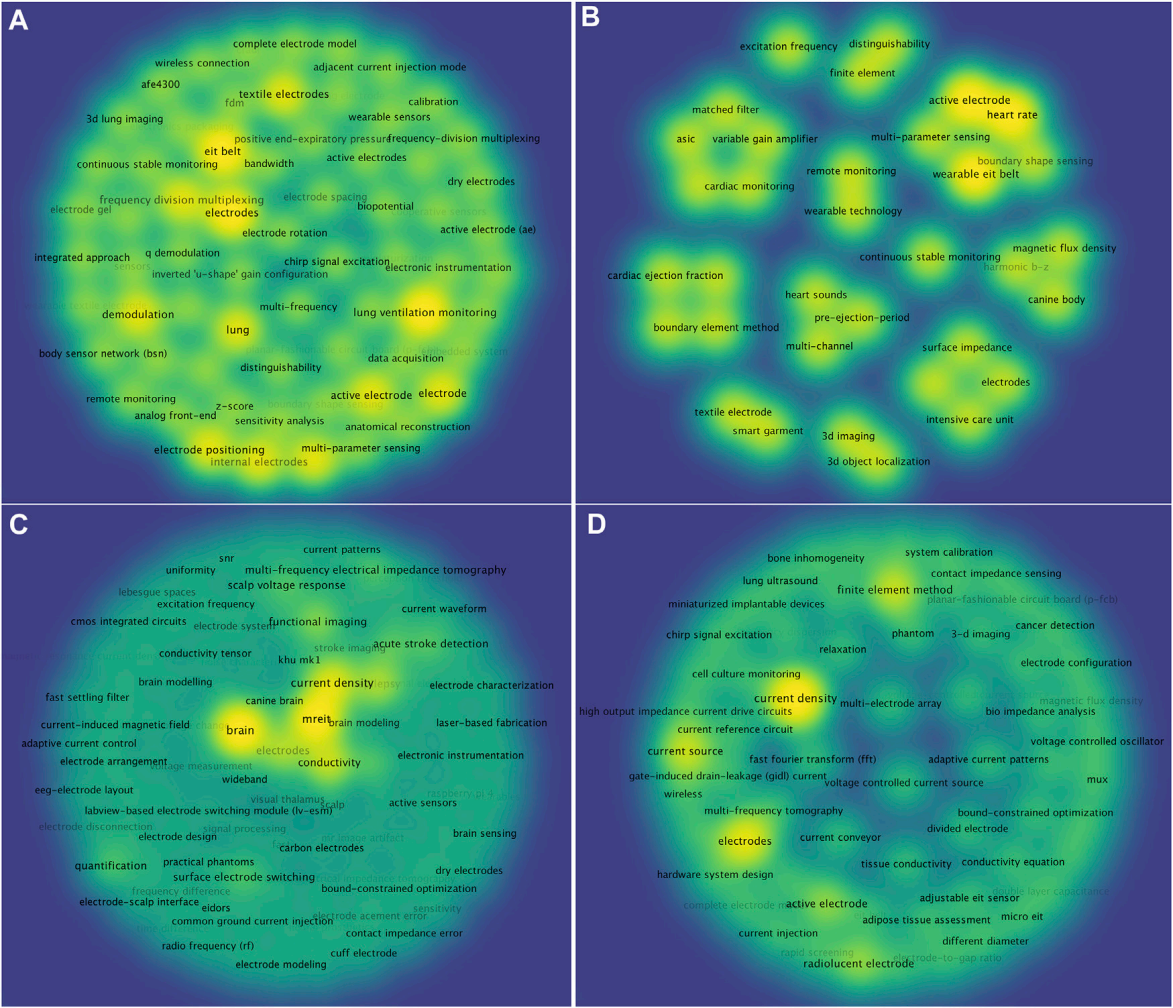
Keywords of EIT hardware research in specific applications as “pulmonary” **(A)** “hemodynamics” **(B)**, “tumor and tissue” **(C)** and “brain” **(D)**. The label indicated keywords, the reduced color from yellow green indicated density of each keyword calculated by frequency of co-occurrence.

Publications on EIT hardware research were further stratified with the specific applications as “pulmonary”, “hemodynamics”, “brain”, “tumor and tissue” according to the terms given in the EIT^2nd^ book (Adler and Holder, 2021). Of the 592 publications, these terms were contained in the abstracts or titles of 285 articles: “pulmonary” 70 (24.6%), “hemodynamics” 20 (7.0%), “tumor and tissue” 112 (39.3%) and “brain” 83 (29.1%). The keywords density visualized map showed that the keywords regarding “voltage”, “current”, “electrode”, “integrated circuit”, “three-dimensional”, “belt”, “wearable”, “wireless”, etc., were shared by all of these applications. Among them, various types of electrodes such as “active electrode”, “electrode position”, “textile electrode”, “electrode gel”, “carbon electrode” etc., had highest occurrence frequency (Figure 6). Moreover, keywords in “pulmonary” and “hemodynamic” applications focused on monitoring such as “ventilation monitoring”, “continuous stable monitoring” and were represented by silhouette recognition such as “anatomical reconstruction”, “matching filters” and dynamic imaging with high-speed such as “frame rate”, “multi-frequency” (Figures 6A,B). While keywords in “tumor and tissue” and “brain” applications focused on diagnosis such as “stoke”, “cancer detection”, “epilepsy” and were represented by optimization of precision such as “fast setting filter”, “rapid screening”, “signal-to-noise ratio” (Figures 6C,D). Topics trend of EIT hardware research in specific research applications were visualized in Supplementary Figure S2.

**FIGURE 7:**
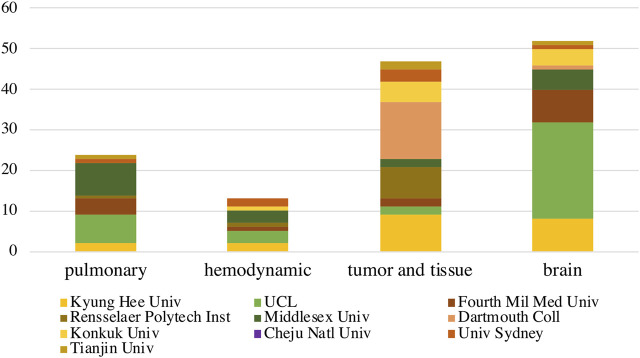
The EIT hardware publications by top 10 institutes on specific applications. The *X*-axis represented application field of “pulmonary”, “hemodynamics”, “tumor and tissue” and “brain”, the *Y*-axis represented the publication proportions listed by publication number. The colour represented institute. The color areas represented the publication number.

Besides, specific applications researches in top 10 institutes were also evaluated. Researches by UCL (7), Middlesex Univ (8), Fourth Mil Med Univ (4) focuses on pulmonary applications, while UCL (3), Middlesex Univ (3) also focuses on hemodynamic applications. Dartmouth Coll (14), Kyung Hee Univ (9), Rensselaer Polytech Inst (8) contributed most to tumor and tissue applications, while the UCL (24), Kyung Hee Univ (8), Fourth Mil Med Univ (8) similarly contributed a lot to brain applications (Figure 7).

### Highly burst keywords on EIT hardware research

**FIGURE 8:**
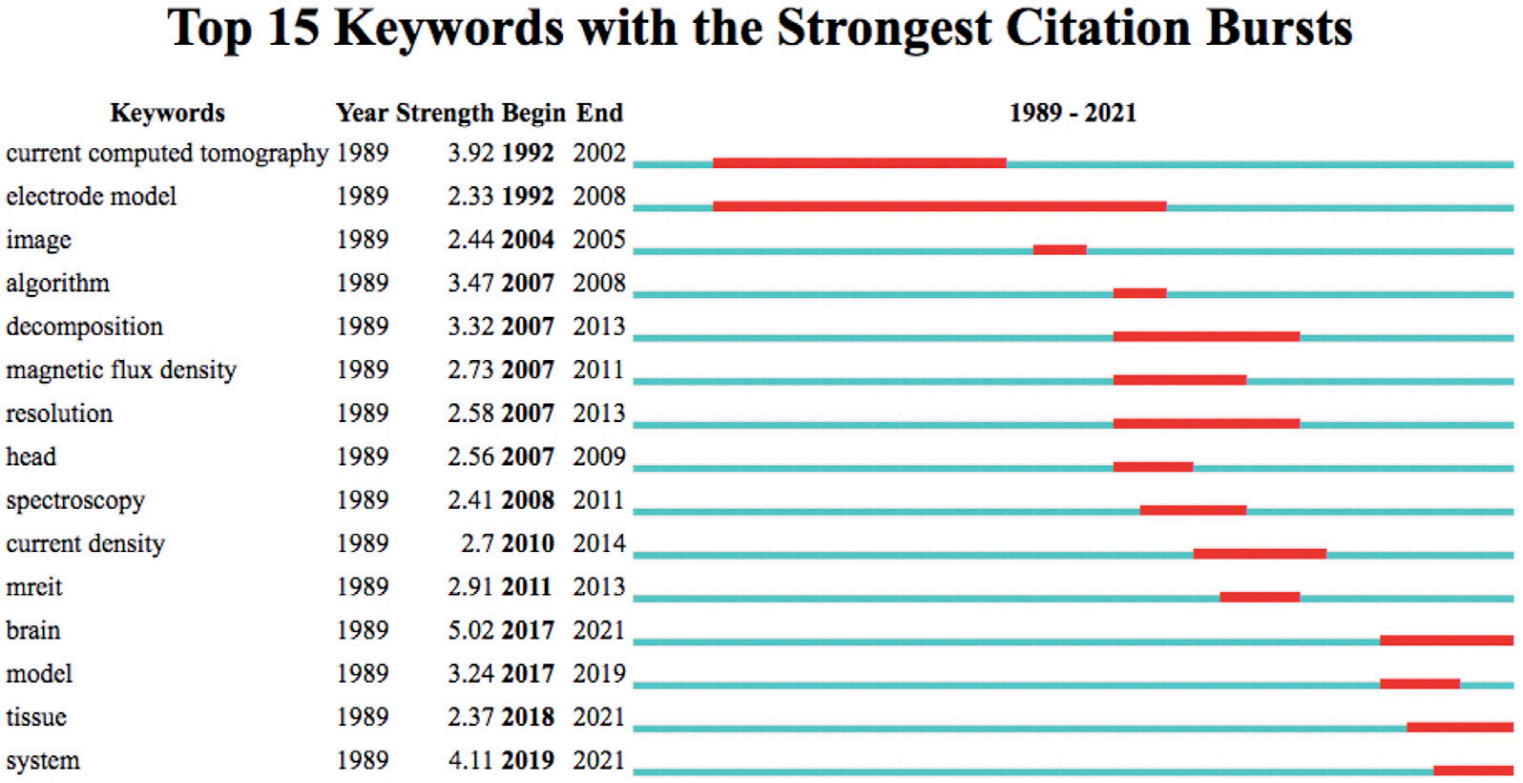
The top 15 keywords with the strongest burst value on EIT hardware research from 1989.01.01 to 2021.12.31. The blue bar indicates the years in which keywords received slight increases in co-occurrence frequency, while the red bar indicates a sharp increase in co-occurrence frequency.

Keywords with strong burst intensity are another essential index reflecting the research hotspots. The top 15 highly burst keywords (described as strength) are summarized in Figure 8. During the period from 1989 to 2021, except for the keyword “current computed tomography”, the keyword “brain” (5.02) had the highest burst value, followed by “system” (4.11), “algorithm” (3.47), “decomposition” (3.32), and “model” (3.24). The keywords “algorithm” (3.47), “decomposition” (3.32) and “magnetic flux density” (2.73) were strongly concerned before 2014, while after 2014, keywords “brain”, “model”, “tissue” and “system” were strongly burst (Figure 6). Overall, the keyword “electrode model” has the longest burst period from 1992 to 2008. Notably, “brain”, “tissue”, and “system” maintained a high burst value until now, indicating that these research directions have received continuous attention in recent years. The top 15 highly burst keywords during separate time periods of 1989–2005 and 2005–2021 are summarized in Supplementary Figure S1.

### Indicators to evaluate the performance of EIT hardware system

**FIGURE 9:**
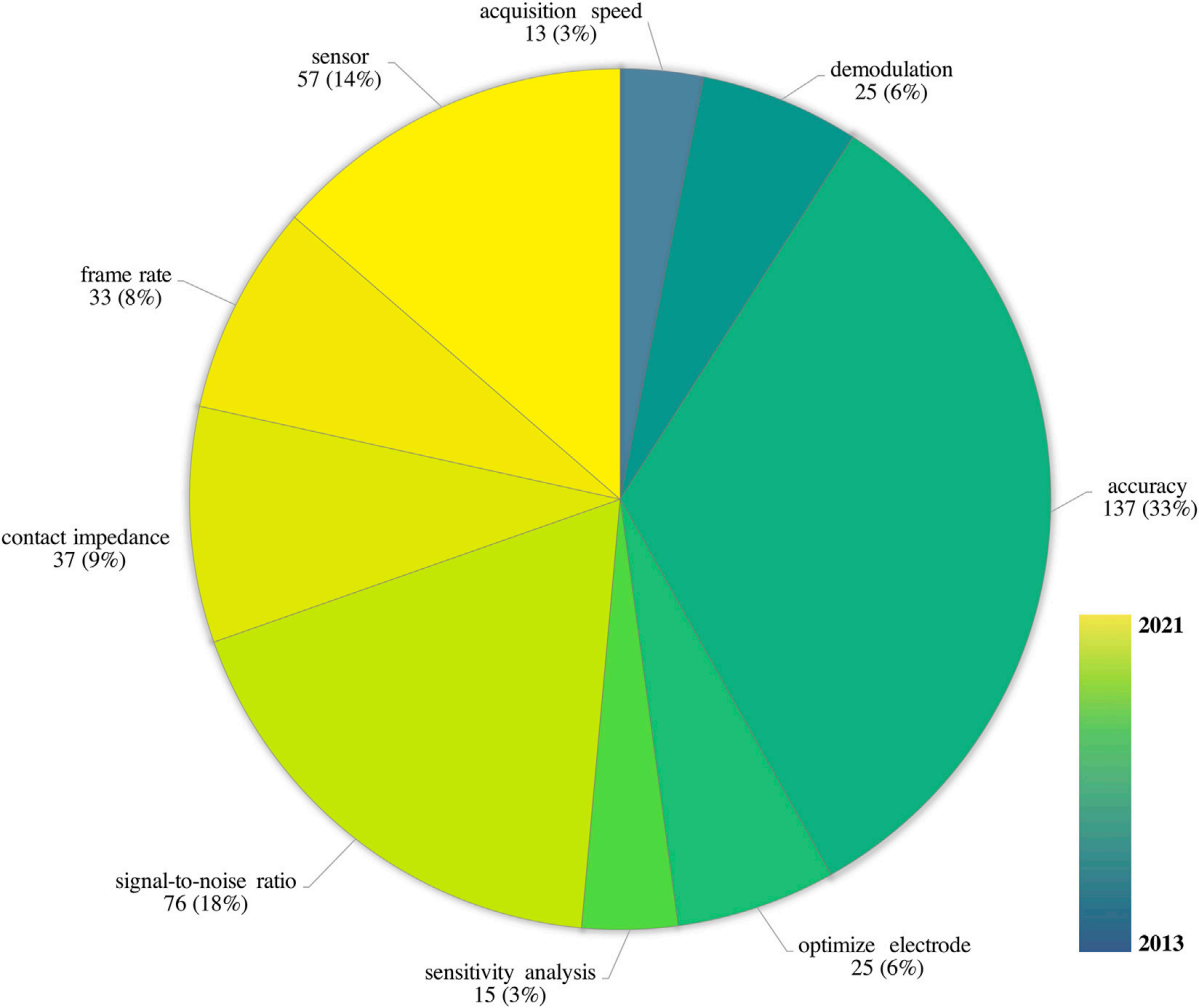
Development of evaluating indicators for EIT hardware performance. The palate color of the pie chart represented each indicator, the palate aera represented the number (percentage%) of each indicator, the gradient color bar from blue to yellow indicated the average year of occurrence M (IQR) for each indicator calculated from the publishing year of the source research.

Performance of EIT hardware system is also critical for EIT hardware development. Publications contained keywords evaluating hardware performance in the title or abstract were analyzed. The most frequent evaluation indicators are accuracy 137 (33%), signal-to-noise ratio 76 (18%) and sensor 57 (14%). The earlier evaluation indicators are acquisition speed [M(IQR) 2013 (2008.5, 2017.5)] and demodulation [2014 (2010, 2018)] which mainly focus on image acquisition.˙Later on, the evaluation indicators of hardware system gradually changed from improving precision such as “sensitivity analysis” [2014 (2008, 2020)], signal-to-noise ratio [2015 (2013.2, 2016.8) ] to optimizing sensitive such as “frame rate [2017 (2015.5, 2018.5) ]”, sensor [2021 (2020, 2022) ] (Figure 9).

## Discussion

In the present study, we provided a quick and objective reference for interested researchers by elaborating the characteristics of EIT hardware-related publications, identifying the influential articles and highlighting research characteristics of specific applications, which provided insights for the most noteworthy research areas and predicted future study directions.

Although the number of publications was less than 50 per year, the increasing trend indicates that the EIT hardware is an ongoing research topic of interest. China, South Korea, UK, USA and Germany have published most of the studies in this field. Cooperation occurred also mainly among the institutions from these countries. For example, research from Korea and UK developed the famous Mark1 EIT system with novel calibration methods for current source and voltmeters (Oh et al., 2007a; Oh et al., 2007b). Recently, an EIT system for dynamic three-dimensional imaging of changes in conductivity distribution in the human head was developed by the researchers in Germany, UK and Thailand (Ouypornkochagorn et al., 2022). These international collaborations demonstrated that system is an important research topic in the field of EIT research.

Academic actualities in the research field of EIT hardware showed that almost all influential EIT research groups have developed their own EIT systems and published the technical details in journals related to biomedical circuits. For instance, the Dartmouth College research group published an EIT system paper in the journal of *IEEE T BIOMED CIRC S*, which details a broadband and miniaturized EIT system for prostate imaging (Rao et al., 2020). The UCL group developed a highly versatile EIT system for brain imaging and its design was published in the journal of *SENSORS* (Avery et al., 2017). The reason why the research groups developed different systems may be that phantom experiments, animal experiments and human experiments rely on the EIT hardware system be carried out. EIT hardware is an indispensable link for EIT research to move from simulation to practical applications. Besides, commercial EIT devices are expensive. Some manufacturers have stopped producing prototypes for lab use. In order to test hypotheses and setting variations, research groups would have to construct their own EIT systems, such as KHU Mark 1, KHU Mark 2.5, Scouse Tom and FMMU 5 (Oh et al., 2007a; Hun et al., 2014; Avery et al., 2017; Shi et al., 2018). These may explain our result that these institutions drilled in different areas of applied research.

Research topic analysis by keywords and reference articles with the high density and citation showed that electrode model, hardware system for 3D imaging, hardware characteristics (broadband, wearability and excitation pattern) were the highest concern topics in the research field of EIT hardware system. In terms of electrode model, which is the topic change over time, after Somersalo *et al.* proved the existence and uniqueness for electrode model in numerical computation of EIT (Somersalo et al., 1992), McAdams et al. discussed the relationship between the performance of EIT hardware and practical factors affecting electrode-gel-skin interface impedance in EIT (McAdams et al., 1996). Subsequently, Vauhkonen *et al.* proposed a finite element-based method based on the complete electrode model, taking the presence of electrode-skin contact impedance measured by EIT hardware into account (Vauhkonen et al., 1999). As for 3D imaging, Cherepenin et al*.* designed the classical 3D EIT system and performed its clinical testing. Additionally, some studies attempted some EIT hardware systems for specific purposes or applications like high-resolution, broadband, breast cancer detection, as well as wearable hand gesture recognition. For example, in applications for “pulmonary” and “hemodynamic”, dynamic imaging with high-speed such as “frame rate”, “multi-frequency” was concerned. While for “tumor and tissue” and “brain”, studies focused on optimizing the system precision to diagnosis disease. These publications with high number of citations proposed solutions to key problems in EIT hardware research.

The earliest keywords that reached the threshold appeared after 2005. In the early years, keywords mainly focused on imaging-related hardware technologies and reconstruction algorithm, such as electrode model (Bayford, 2006; Adler and Boyle, 2017)and b-z algorithm (Park et al., 2004; Seo and Woo, 2011). This result indicated that the purpose of early research on EIT hardware was to improve the EIT image quality [‘contrast’ and ‘distinguishability’ (Adler et al., 2011)] through the cooperation of hardware (‘electrode model’ (Mueller et al., 1999)) and algorithm (‘state estimation’ (Kolehmainen et al., 2001) and ‘decomposition’ (Clay and Ferree, 2002)). Recent keywords mainly focus on the performance improvement of each module of EIT hardware system by applied new designs or techniques, including ‘voltage measurement’ (Fu et al., 2022; Shi et al., 2022), current source (‘Howland current source’ (Oh et al., 2011), ‘current driver’ (Hong et al., 2010; Shahghasemi, 2020)and ‘output impedance’ (Shishvan et al., 2021)) and novel electrode sensor (‘textile electrodes (Katashev et al., 2018; Hu et al., 2021)’ and ‘active electrode’ (Wu et al., 2019)). For example, Saulnier *et al.* and Liu *et al.* designed a DSP-based current source and a FPGA-based adaptive different current source in order to meet the requirement of high output impedance for current source in the most popular EIT hardware frameworks with a parallel and multiple source architecture (Saulnier et al., 2020; Liu et al., 2021). The shift in focus from early keywords to recent keywords showed that the research trend of EIT hardware is to improve the overall performance of EIT system by applying advanced circuit strategies to modify various existing systems, such as the frequency band, current excitation method, output impedance of current source and frame rate.

Since the introduction of the first systems in the early 1980s, EIT instruments have evolved with advances in analog and digital electronics. EIT instruments are designed primarily using analog techniques and processing the digital domain, with extensive use of digital signal processors and programmable logic devices (Bayford and Polydorides, 2019). Our analysis shows some of the components that are combined to produce EIT instruments in order to optimize the direction of a particular system, such as application drivers, algorithms, voltages, reconstructions, etc. Additionally, it is possible that novel imaging modalities by means of combining EIT and other imaging techniques will continue to be developed for improving the practicality of EIT (Goss et al., 2003).

This is one of bibliometric analysis series we performed regarding EIT. A total of 733 papers were identified with the searching keywords but among them, 141 papers rather focused on algorithms or clinical applications. The percentage of inappropriate topic (19.2%) is much higher than the other bibliometric analysis we conducted on EIT algorithm and clinical applications. Even in the 592 papers we decided to keep for the analysis, some also contain contents regarding algorithms and/or clinical applications. The decision was hard to make whether a study should be considered as hardware related and included to the current analysis. We could not develop a hard rule or cut-off threshold to separate hardware related studies from algorithm and application studies. Therefore, in the hot topics and top keywords, the readers may find a lot of algorithm and application related terms. This could be considered as a limitation of the study (Gonzalez de Dios et al., 1997; Hicks, 2016). On the other hand, EIT systems are built on hardware and software, with specific algorithm and application directions. Therefore, it might not be reasonable to exclude studies with those aspects in them.

## Conclusion

To sum up, research topics on EIT hardware spread out in all fields involved in EIT, electrode development was research focus through the years. System performance, particularly in the areas of system bandwidth and precision in applications may be the future directions of hardware research.

## Data Availability

The original contributions presented in the study are included in the article/Supplementary Material, further inquiries can be directed to the corresponding authors.
