# Supplementary material for: Characteristics and topic trends on electrical impedance tomography hardware publications
Source: Front Physiol. 2022 Oct 13;13:1011941. doi: 10.3389/fphys.2022.1011941 (PMC9608147; doi:10.3389/fphys.2022.1011941)
Supplement: Supplementary file 1 [file Table1.DOCX]

**Supplementary material**

Supplementary Figures

Figure S1 The top 15 highly burst keywords during separate time periods of 1989–2005(left) and 2005-2021(right) on EIT hardware research from 1989.01.01 to 2021.12.31.

Figure S2 Visualization map of keywords time trend on EIT hardware researches in specific applications.

Supplementary Tables

Table S1 Merge of keywords on EIT hardware research

Table S2 Top 10 publications with high citations on EIT hardware research

Table S3 Major keywords of each co-occurrence cluster* on EIT hardware research

**Supplementary Figures**


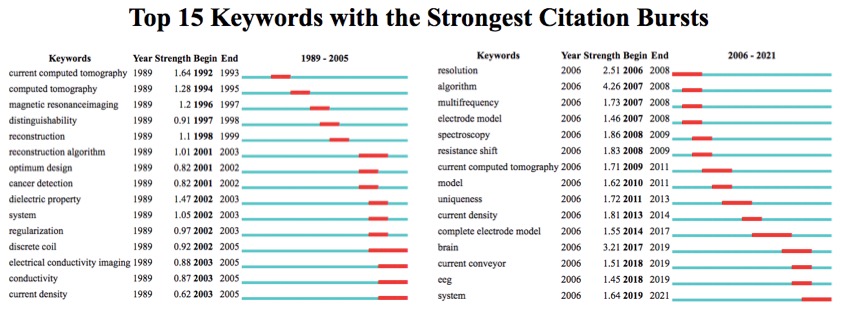


**Figure S1**| The top 15 highly burst keywords during separate time periods of 1989–2005(left) and 2005-2021(right) on EIT hardware research from 1989.01.01 to 2021.12.31. The blue bar indicates the years in which keywords received slight increases in co-occurrence, while the red bar indicates a sharp increase.

**
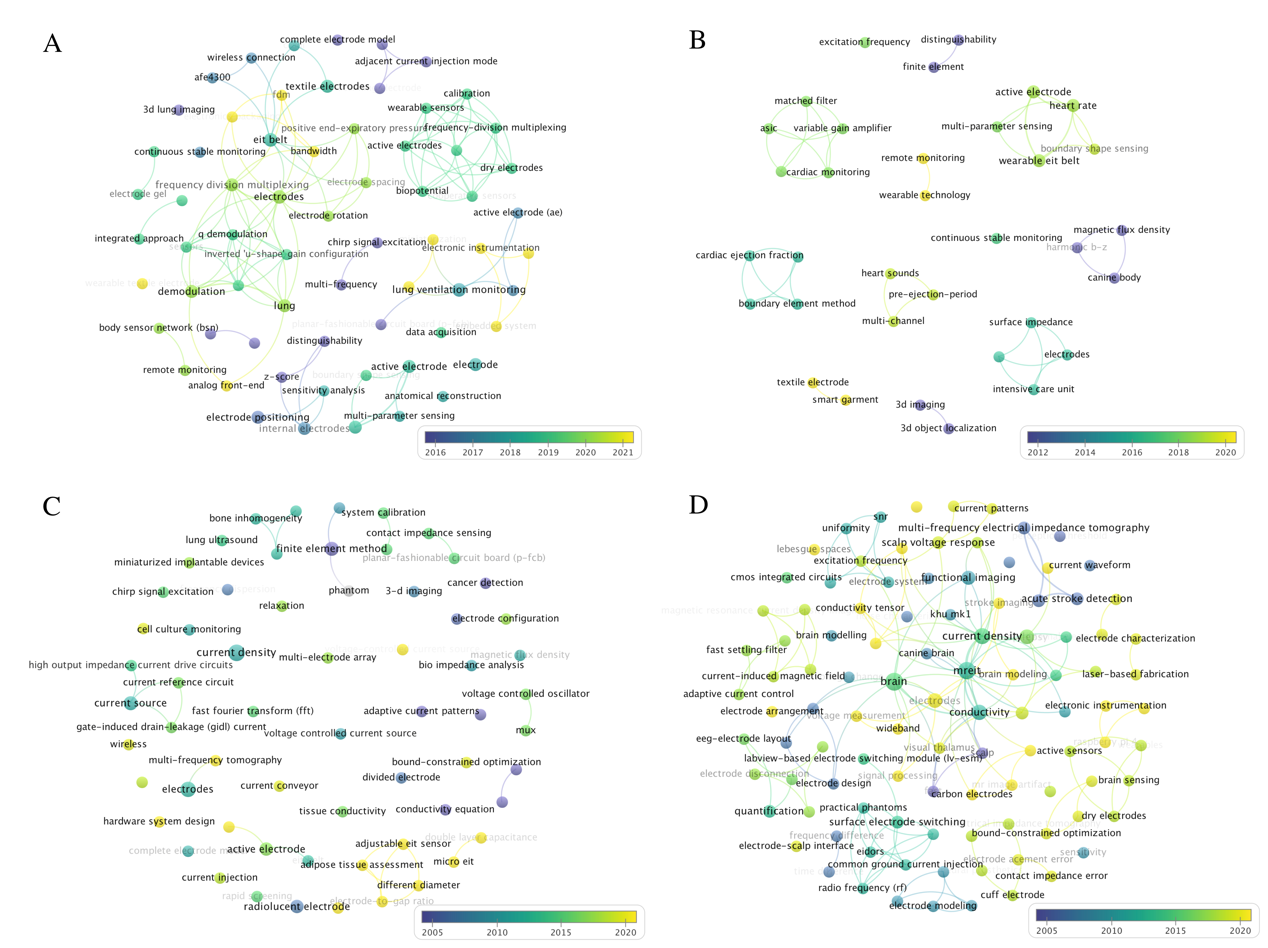
**

Figure S2 Visualization map of keywords time trend on EIT hardware researches in specific applications. The label indicates keywords in research applications of “pulmonary” (A), “hemodynamics” (B),“tumor and tissue” (C) and “brain” (D), The color indicates average publication year. The circle size indicates frequency of co-occurrences. The line thickness indicates link strengths.

**Supplementary Tables**

**Table S1** Merge of keywords on EIT hardware research

| Merge to | label |  | Merge to | label |
| --- | --- | --- | --- | --- |
| electrical impedance tomography | eit, tomography, electrical impedance, electric impedance tomography, electrical-impedance tomography, electrical impedance tomography (eit), impedance tomography |  | mreit | magnetic resonance electrical impedance tomography, magnetic resonance electrical impedance tomography (mreit), mr-eit, magnetic resonance imaging |
| current  source | current sources, current density imaging, current density imaging (cdi), current-density |  | conductivity | conductivity image, electrical conductivity |
| image reconstruction | image-reconstruction, images, impedance imaging, reconstruction |  | Breast  cancer | breast cancer detection, breast-cancer detection |
| bioimpedance | impedance, bio-impedance |  | head | human head |
| contact impedance | contact impedances |  | tissue | tissues |
| eit belt | electrode belt |  | model | models, modelling |
| electrodes | electrode |  | sensor | sensors |
| inverse problem | inverse problems |  | brain | brain imaging |

**Table S2** Top 10 publications with high citations on EIT hardware research

| Rank | Title | Publication Type | Author | Publication Year | journal | Citations |
| --- | --- | --- | --- | --- | --- | --- |
| 1 | Existence and uniqueness for electrode models for electric current computed tomography. | Article | Somersalo, E | 1992 | SIAM J. APPL. MATH | 585 |
| 2 | Bioimpedance Tomography (Electrical Impedance Tomography) | Review | Bayford RH | 2006 | ANNU REV BIOMED ENG | 259 |
| 3 | Three-dimensional  electrical impedance tomography based on the complete electrode model | Article | Vauhkonen PJ | 1999 | IEEE T BIO-MED ENG | 245 |
| 4 | A 3D electrical impedance tomography (EIT) system for breast cancer detection | Article | Cherepenin, V | 2001 | PHYSIOL MEAS | 156 |
| 5 | Magnetic resonance  electrical impedance tomography (MREIT) for high-resolution conductivity imaging | Review | Woo EJ | 2008 | PHYSIOL MEAS | 154 |
| 6 | A broadband high-frequency electrical impedance tomography system for breast Imaging | Article | Halter, RJ | 2008 | IEEE T BIO-MED ENG | 123 |
| 7 | Factors affecting electrode-gel-skin interface  impedance in electrical impedance tomography | Review | McAdams, ET | 1996 | MED BIOL ENG COMPUT | 105 |
| 8 | Three-dimensional EIT  imaging of breast tissues: System design and clinical testing | Article | Cherepenin V | 2002 | IEEE T MED IMAGING | 104 |
| 9 | Assessment of errors in static electrical impedance tomography with adjacent and trigonometric current patterns | Article | Kolehmainen, V | 1997 | PHYSIOL MEAS | 102 |
| 10 | Towards a high accuracy wearable hand gesture recognition system using EIT | Proceedings Paper | Wu Y | 2018 | IEEE ISCAS(2018) | 99 |

**Table S3** Major keywords of each co-occurrence cluster* on EIT hardware research

| co-occurrence cluster  (label) | keywords |
| --- | --- |
| Cluster 1:  (MREIT principle) | Image reconstruction; mreit; current density; one-component; resolution; b-z algorithm; decomposition; uniqueness; flux density; j-substitution algorithm; magnetic flux density; optimization; contrast |
| Cluster 2：  (EIT system design) | bioimpedance; multi-frequency; calibration; current source; tissue; dielectric-properties; impedance spectroscopy; active electrode; biomedical instrumentation; howland current source; output impedance; |
| Cluster 3:  (data acquisition strategy) | eit system; sensor; validation; fpga; voltage measurement; data acquisition; flow; internal electrodes; current driver; |
| Cluster 4:  (hardware related to image reconstruction ) | inverse problem; distinguishability; simultaneous reconstruction; finite element method; regularization; electrode models; boundary; internal electrical-properties; optimal current patterns; state estimation; |
| Cluster 5:  (Electrode system) | electrodes; contact impedance; sensitivity; impedance measurement; eit belt; skin; textile electrodes; |
| Cluster 6:  (brain application platform) | phantom; head; brain; density; eidors; stroke; brain-function; epilepsy; practical phantom; stimulation; |
|  |  |

* keywords co-occurrence frequency >5
